# The association between risk perceptions, anxiety, and self-reported changes in tobacco and nicotine product use due to COVID-19 in May-June 2020 in Israel

**DOI:** 10.1186/s12889-023-15351-1

**Published:** 2023-04-25

**Authors:** Noah G. Rubinson, Geoffrey T. Fong, Shannon Gravely, Anne C. K. Quah, Michal Bitan, Shahar Lev Ari, Laura J. Rosen

**Affiliations:** 1 School of Medicine, Sackler Faculty of Medicine, Tel Aviv University, Ramat Aviv, Israel; 2 Department of Psychology, University of Waterloo, Waterloo, Canada; 3 School of Public Health Sciences, University of Waterloo, Waterloo, Canada; 4 Ontario Institute for Cancer Research, Toronto, Canada; 5 Department of Computer Science, College of Management Academic Studies, Rishon LeZion, Israel; 6 Department of Health Promotion, School of Public Health, Sackler Faculty of Medicine, Tel Aviv University, Ramat Aviv, Israel

**Keywords:** COVID-19, Tobacco use, Smoking, Nargila, Electronic cigarettes, IQOS, Home smoking, Cultural differences

## Abstract

**Background:**

Early in the COVID-19 pandemic, reports about a possible protective effect of nicotine on COVID-19 conflicted with messaging by public health organizations about increased risks of COVID-19 due to smoking. The ambiguous information the public received, combined with COVID-19-induced anxiety, may have led to changes in tobacco or other nicotine product use. This study examined changes in use of combustible cigarettes (CCs), nargila (hookah/waterpipe), e-cigarettes, and IQOS and home-smoking behaviors. We also assessed COVID-19 related anxiety and perceptions regarding changes in risk of COVID-19 severity due to smoking.

**Methods:**

We used cross-sectional data from a population telephone survey that was conducted in Israel in the early phase of the COVID-19 pandemic (May–June 2020) and included 420 adult (age 18+) individuals who reported having ever used CCs (*n* = 391), nargila (*n* = 193), and/or electronic cigarettes (e-cigarettes)/heated tobacco products (e.g., IQOS) (*n* = 52). Respondents were asked about the effect that COVID-19 had on their nicotine product use (quit/reduced use, no change, increased use). We assessed changes in product use, risk perceptions, and anxiety using adjusted multinomial logistic regression analyses.

**Results:**

Most respondents did not change their frequency of product use (CCs: 81.0%, nargila: 88.2%, e-cigarettes/IQOS: 96.8%). A small percentage either decreased use (CCs: 7.2%, nargila: 3.2%, e-cigarettes/IQOS:2.4%) or increased use (CCs:11.8%, nargila:8.6%, e-cigarettes/IQOS:+ 0.9%). 55.6% of respondents used a product in the home prior to COVID-19; but during the first lockdown COVID-19 period, a greater percentage increased (12.6%) than decreased (4.0%) their home use. Higher levels of anxiety due to COVID-19 were associated with increased home smoking (aOR = 1.59, 95% CI:1.04–2.42, *p* = 0.02). Many respondents believed that increased severity of COVID-19 illness was associated with CCs (62.0%) and e-cigarettes/vaping (45.3%), with uncertainty about the association being lower for CCs (20.5%) than for vaping (41.3%).

**Conclusions:**

While many respondents believed that nicotine product use (particularly CCs and e-cigarettes) was associated with increased risk of COVID-19 disease severity, the majority of users did not change their tobacco/nicotine use. The confusion about the relationship between tobacco use and COVID-19 calls for clear evidence-based messaging from governments. The association between home smoking and increased COVID-19-related stress suggests the need for campaigns and resources to prevent smoking in the home, particularly during times of stress.

**Supplementary Information:**

The online version contains supplementary material available at 10.1186/s12889-023-15351-1.

## Implications

The initial phase of the COVID-19 pandemic (March until June 2020) in Israel appears to have had no net impact on tobacco use behaviors among individuals who had ever smoked, vaped, or experimented with tobacco or nicotine. It should be noted that no net impact is a less negative outcome than the typical increase of smoking observed following other population crises, such as natural disasters and the 9/11 attack. However, home-smoking increased during the first lockdown period, increasing the already high prevalence (56%) of home smoking in Israel. Increased COVID-19-related anxiety was associated with increased home smoking.

## Introduction

COVID-19 is a population-level stressor of unprecedented global proportions due to its impact on health and the economy. The pandemic led to significant psychological trauma (e.g., stress, anxiety, depression) as a result of sudden lifestyle changes and uncertainty about the future. The United States Centers for Disease Control and Prevention (US CDC) and the World Health Organization (WHO) identified populations at increased risk of severe illnesses from COVID-19: those older than 65 years; those with underlying medical conditions, such as chronic lung disease, diabetes, cardiac disease, cancer; and those with compromised immune systems. The CDC also identified those who smoke (or have a history of smoking) as being at an increased risk for severe complications from COVID-19 [1]. There was little to no information about the association of vaping on the severity of COVID-19.

While the data are not conclusive, a number of studies have reported that smoking increases the risk of greater disease severity and mortality from COVID-19 [2, 3]. Other studies found the opposite—that disease severity and hospitalization rates were lower for smokers relative to non-smokers [4–6]. Early reports from China [7], the US [8], Italy [9], France [10], and Germany [11] showed that smokers were underrepresented among hospitalized patients with COVID-19. This finding provoked substantial debate about the relationship between smoking and COVID-19 in the medical and lay press [7, 12–15]. Some scientists rejected the findings on the basis of study flaws and data limitations, and others considered whether a possible protective influence of nicotine might exist [13, 16]. Other meta-analyses emerged with contradictory results, reporting that current and former smokers were at increased risk of significant disease severity compared to never smokers [15, 17, 18].

A substantial body of evidence demonstrates that health communications have a significant impact on tobacco-related behavior [19–21]. With the onset of COVID-19, media coverage of all aspects of the pandemic in Israel was frequent. The relationship between smoking and COVID-19 received substantial attention. In addition to the international reports, two large local studies based on existing population-level data on smoking status prior to COVID-19 received wide coverage. The first, based on data from Israel’s largest Health Maintenance Organization (HMO), Clalit Health Services, which serves 3 million adults, found that the risk of transmitting COVID-19 was reduced by half among current smokers [22]. The second, based on the complete medical records of 4353 individuals who were members of the HMO, found that smoking did not significantly increase disease severity among verified COVID-19 patients [23]. However, a quantitative content analysis of 11 leading media sites, which did not differentiate between increased risk for COVID-19 and increased severity of COVID-19 found that 86.6% of the 82 included articles described an increase in COVID-19 risk among tobacco users, while 3.6% of articles described a decrease in COVID-19 risk among tobacco users [24].

Concern was raised among health professionals that media reports of a possible advantage to smokers might cause some individuals to lose motivation to quit or even increase their smoking [25]. As the scientific debate raged, some governmental and health organizations provided clear messages that smoking was a risk factor for COVID-19 transmission or for a more severe case of COVID-19. For example, the Israel Ministry of Health’s website section entitled “Smoking as a risk factor for coronavirus” included the statement: “Inhalation of smoke or vapors into the lungs …. constitutes a risk factor for becoming infected with coronavirus or for complications of the virus, both for smokers and for those in their immediate surroundings [26]. The WHO was more moderate, stating that: “At the time of preparing this Q&A, there are no peer-reviewed studies that have evaluated the risk of SARS-CoV-2 infection associated with smoking. However, tobacco smokers (cigarettes, waterpipes, bidis, cigars, heated tobacco products) may be more vulnerable to contracting COVID-19″ [27].

From the onset of the pandemic, multiple studies examined smokers’ behavioral changes across several countries. Findings have generally shown that smokers have had varying behavioral reactions, ranging from quitting to increasing their consumption of cigarettes. Generally, however, most smokers appear to have not made substantial changes to their consumption patterns [28]. An online social media survey conducted in Israel among current and ex-smokers found that 7% of respondents quit during the first lockdown period, 44.4% of current smokers increased their cigarette consumption, and 16% attempted to quit [29]. The study however did not assess changes in nargila use, electronic-cigarettes (e-cigarettes), or IQOS. Further, it was conducted only in Hebrew, on social media sites, and it is unclear whether the Arab population participated at all. In Israel, Arabs (Muslims, Christians, and Bedouins) comprise about a fifth of the population. Inclusion of major subpopulations in research is critical to get reliable population-wide estimates. This is especially true regarding smoking, as different population groups have different underlying smoking behaviors and may respond to crises differently. Persistent differences in the smoking behavior of the Jewish and Arab populations have been observed in Israel for decades; these include differences in: smoking prevalence (2020 Overall: 20.1%, Jewish male: 22.6%, Jewish female:15.8%, Arab male:38.2%, Arab female:10.2% [30]); patterns of tobacco and nicotine product initiation [31]; child exposure to tobacco smoke [32]; and adolescent exposure to tobacco smoke [33]. We investigated changes in use of combustible cigarettes (CCs), nargila (hookah/waterpipe), e-cigarettes/IQOS (the only heated tobacco product sold in Israel) due to COVID-19 in our analysis. We used a sample drawn randomly from a nationally representative database covering the entire Israeli population. The survey was conducted in Hebrew, Arabic, Russian, and English. We also examined changes in home-smoking behaviors, COVID-19 related anxiety and perceptions regarding changes in risk of COVID-19 severity due to smoking.

## Methods

In Israel, the first case of COVID-19 was diagnosed on February 21, 2020. Social distancing regulations began on March 11, 2020, and were quickly expanded to include school and other closures. A national emergency was declared on March 19, 2020, followed by restrictions on movement and directives to remain within 100 m of one’s home. On May 4, 2020 a plan to gradually ease restrictions was passed by the government, with most restrictions relaxed by May 20, 2020 [34, 35]. We conducted a cross-sectional study between May 14 and June 22, 2020, during and following the end of the first COVID-19 lockdown in Israel.

### Sampling strategy and weighting

Our sampling frame was comprised of a nationally representative sample of adult (aged 18+) Israeli residents. The sample of 2500 individuals was obtained from a database held by the Israel Central Bureau of Statistics (CBS), and was representative in terms of distributions of age, sex, population group (Jews and Others, Arabs), and geographic area. Phone numbers were dialed six times before they were deemed unresponsive. Participation was voluntary (respondent provided verbal informed consent), and one telephone interview was conducted per household. The interview lasted 10 minutes on average, and was offered in Hebrew, English, Arabic, or Russian.

Weighting was performed based on the distribution of population groups (Jews and Others, Arabs), sex (male/female), and age groups (18–39, 40–59, 60+). Population distributions for current and former smokers (including users of CCs, pipes, cigars, and nargila) were obtained from the 2017 Social Survey conducted by the Central Bureau of Statistics (CBS) of Israel, using the Table Generator function on the website [36]. Because the CBS data included persons aged 20+, and our data included persons 18+, we adjusted the weights from numbers obtained from the CBS by adding 10% to the raw numbers in the youngest age group (e.g., 18–39). Twelve cell weights were calculated, for each combination of population group, sex, and age category, by dividing the cell percent from the CBS data by the cell percent from the data in the current survey. Details can be found in Supplementary File 1.

### Inclusion criteria

Study participants were asked: “Have you ever smoked a cigarette, or used nargilla, or tried an e-cigarettes such as JUUL, or any other tobacco or nicotine vaporizing product, such as IQOS, even just one puff? Those who were aged 18 or over, and reported having ever used one of these products were eligible for inclusion in this current study.

### Measures

#### Population Group

We defined population group in the manner done by the CBS [37]: we used a question on religion (Jewish/Muslim/Christian/Druze/Other) to define population group by creating an indicator for Jews and Others (“Jewish population”) or Arabs (“Arab population”, e.g., Muslim/Christian/Druze). Standard presentation of information on smoking in Israel is according to population group by sex [38].

#### Socio-demographic variables

Standard questions were used to assess sex, age, religion, marital status, and income [39]. We also asked respondents whether they had children living with them in their home.

#### Smoking and nicotine product use

Questions about smoking were based on the International Tobacco Control Policy Evaluation (ITC) Project surveys [40]. We asked: “On average, how often did you smoke cigarettes?” Possible responses were: “Daily/Almost Daily/Most days”; “Less than daily, but at least weekly”; “Less than weekly, but at least monthly”; “Less than monthly; former, not at all”. We used this to create the variable “current smoker” (those who currently smoke at least monthly, yes/no), and “ever smoker” which included experimenters, anyone who reported smoking less than monthly, and former smokers. We asked whether respondents had ever smoked nargila, with possible answers: “Experimented”; “Former”; “Current”; “Never”. We categorized the answers into current (yes/no) and ever (yes/no). Regarding e-cigarettes/IQOS use, we asked: “Have you ever vaped, that is, used an e-cigarette such as JUUL for example, or any other tobacco or nicotine vaporizing product, such as IQOS for example, even just once?” Possible answers were: “Experimented”; “Former”; “Current”; “Never”. We categorized the answers into current (yes/no) and ever (yes/no).

#### Change in product use due to COVID-19

Questions were modified from COVID-19 questions used in the ITC surveys in the United States, Canada, England, and Australia. We asked: “What effect has the coronavirus outbreak had on your smoking?” Response options were: “Because of it, I quit smoking”; “Because of it, I’m thinking of quitting smoking”; “Because of it, I’m smoking less”; “Because of it, I’m smoking more”; “Because of it, I started smoking again, even though I had quit before”; “Because of it, I started smoking”; “It has had no effect at all on my smoking”. Questions regarding change of use in CCs, nargila, and e-cigarettes/IQOS were categorized into three categories: “Less (decreased)”; “Same (no change)”; or “More (increased)”. Responses of “Attempted to quit” were set to missing (CCs: *n* = 3; Nargila: *n* = 1; Vaping: *n* = 1) as we could not determine whether they had changed their cigarette consumption or not. In the event that a quitter returned to using a product, they were categorized as “More (increased)”.

#### Risk perception questions

These questions, also taken from the ITC surveys, explored the relative severity of COVID-19 for smoking and vaping individuals relative to same-age non-smokers/non-vapers. Regarding smoking, we asked: “Thinking about smokers in general, if a smoker got the coronavirus, how severe do you think the illness would be for them, compared to non-smokers of the same age who got it?” Answers were given on a severity scale ranging from 1 “A lot more severe” to 5 “A lot less severe”, with an additional “Don’t know” option. A parallel question was asked to those who reported having ever used an e-cigarette.

#### Changes in anxiety levels due to COVID-19

We asked participants: “How has the coronavirus outbreak and subsequent lifestyle changes have caused you to feel?” Responses were on a 5-point scale ranging from 1 “significantly less anxious than before corona”, to 5 “significantly more anxious than before corona” [41].

*Smoking in the home* was based on a question adapted from the ITC Surveys [42]. We asked all respondents “Before the coronavirus outbreak, how frequently did you smoke (cigarettes, nargila, or electronic cigarettes, or any other tobacco or nicotine vaporizing product) inside your home, including on your balcony or porch?” Response options were: “Daily, more than once a day”; “Daily, once a day”; “Weekly”; “Monthly”; “Less than once a month”; “Never”. We then asked: “How has the coronavirus affected how often you smoke inside the home (cigarettes, nargila, e-cigarettes, or any other tobacco or nicotine vaporizing product) including on your porch? The possible responses were: “More inside the house” “Less inside the home” or “The same amount inside the home”. In this manuscript, “home smoking” refers to use of cigarettes, nargila, e-cigarettes, or any other tobacco or nicotine vaporizing product in the home.

### Statistical analysis

All statistical analyses were conducted using SAS Version 9.4. With the exception of unweighted distributions of variables at baseline, which are presented in Table 1 and described in the Results Section, we present results based on weighted data. We present the adjusted odds ratios (aOR) in Table 2 and in the Results Section.

First, we examined use of all products descriptively. Then, we examined changes in behavior due to COVID-19. Changes were calculated for those who reported ever using a particular product (CCs, nargila, e-cigarettes/IQOS). Analyses of changes in home tobacco use (CCs, nargila, e-cigarettes/IQOS) were conducted on all participants. Multinomial logistic regression was used to examine changes in CC use, nargila use, e-cigarette/IQOS use, and home smoking due to COVID-19. We adjusted for sex, population group, age category, and increases in anxiety due to COVID-19 in all models. We also included current CC use and CC risk perceptions in the CC change model, current nargila use in the nargila change model, current e-cigarette/IQOS use in the e-cigarette/IQOS model, and CC risk perceptions and any current use in the home smoking model. Results of the model for changes in use of e-cigarettes/IQOS are not presented because of convergence failure (due to small sample sizes).

Our analyses examined: (a) risk perceptions regarding the perceived severity of COVID-19 infection for smokers versus non-smokers; (b) risk perceptions regarding the perceived severity of COVID-19 infection in non-smoking vapers versus non-smoking non -vapers; and (c) increased anxiety levels due to COVID-19. We present frequencies of all responses including “don’t know”, and the results of the linear model which included five categories of perceived severity, without the “don’t know” option. We used linear models (Proc GLM) to examine the influence of sex, population group, age category, and anxiety on risk perceptions regarding CCs and e-cigarettes. Current CC use was included in the CC risk perceptions model and current e-cigarette/IQOS use in the model of e-cigarette risk perceptions. We used a linear model to examine the influence of sex, subpopulation, age category, and current CC use on anxiety.

## Results

### Participation

Of the 2500 potential respondents in the initial sampling frame, 361 were disconnected and 211 were deemed unresponsive after being called six times. Of the 1928 households we were able to reach, 792 were ineligible because they were never users of any product, a further 25 were deemed ineligible due to the respondent being under aged 18, and six were unable to answer in any of the four languages of the survey. The tracking of 18 calls was lost due to mistaken or incomplete data collection. Of the 1087 that remained, 667 individuals refused to participate. It is not clear whether those individuals were eligible for the study. We completed a total of 420 interviews. The response rate was 64.2% [43].

### Demographics

The full sample was comprised of 420 respondents. Of the 391 respondents for whom we had information on subpopulation, 77.8% (*n* = 304) were from the Jewish sector and 22.2% (*n* = 87) were from the Arab sector. Among respondents, 35.4% (*n* = 138) were female and 64.6% (*n* = 252) were male. The sex distribution differed between the populations (*p* < 0.001), with Arab women slightly overrepresented relative to population figures of ever smokers from the CBS (Arab men: 87.4%, *n* = 76; Arab women: 12.6%, *n* = 11). Average age was 48.8 years (Standard deviation: 14.8). 72.6% (*n* = 284) of our participants identified as Jewish, 18.4% (*n* = 72) as Muslims, 3.8% (*n* = 15) as Christians, and 5.1% (*n* = 20) as Other. Most (72.9%, *n* = 288) were married or partnered. Most (61.4%, *n* = 194) reported that they were in average or high income categories, while a minority (38.6%, *n* = 122) reported that they were in income categories below average. Most (60.9%, *n* = 230) reported that children lived with them in their home see Table 1.

**Table 1:** Demographic characteristics of respondents, by changes in cigarette smoking (Raw numbers)

Unweighted % (N)		Less/Quit	Same	More	Overall Sample	***P*** Value
**Population Subgroup**	Jewish and Other Arab	7.7% (23) 2.4% (2)	81.6% (244) 89.4% (76)	10.7% (32) 8.2% (7)	77.9% (299) 22.1% (85)	0.19
**Sex**	Female Male	10.7% (15) 3.9% (10)	77.9% (109) 87.2% (225)	11.4% (16) 8.9% (23)	35.2% (140) 64.8% (258)	0.004
**Age Category**	18–39	7.8% (8)	75.7% (78)	16.5% (17)	27.3% (103)	0.003
	40–59 60+	8.0% (14) 3.1% (3)	82.4% (145) 92.9% (91)	9.7% (17) 4.1% (4)	46.7% (176) 26.0% (98)	
**Religion**	Jewish Muslim Christian Other	8.2% (23) 2.9% (2) 0.0% (0) 0.0% (0)	80.7% (225) 88.6% (62) 93.3% (14) 95.0% (19)	11.1% (31) 8.6% (6) 6.7% (1) 5.0% (1)	72.7% (279) 18.2% (70) 3.9% (15) 5.2% (20)	0.29
**Marital/Partnership Status**	Married/Living with Partner	7.4% (21)	82.7% (234)	9.9% (28)	72.9% (283)	0.43
	Divorced Separated Widowed Single	2.3% (1) 0.0% (0) 9.1% (1) 4.1% (2)	90.7% (39) 100.0% (2) 90.9% (10) 83.7% (41)	7.0% (3) 0.0% (0) 0.0% (0) 12.2% (6)	11.1% (43) 0.5% (2) 2.8% (11) 12.6% (49)	
**Income (Relative to Average Monthly Income of NIS 14,800)**	Lower Average/Higher	6.7% (8) 6.3% (12)	84.0% (100) 82.2% (157)	9.2% (11) 11.5% (22)	38.4% (119) 61.6% (191)	0.74
**Are there children living with you**	Yes	7.5 (17)	81.9% (186)	10.6% (24)	61.2% (227)	0.25
	No	4.9 (7)	86.1% (124)	9.0% (13)	38.8% (144)	

### Behavior: use of CCs, nargila, e-cigarettes/IQOS, and home smoking

In this population of ever-users (current and former users combined) of CCs, nargila, e-cigarettes and/or IQOS, the most common currently used product was CCs (51.0%), followed by nargila (11.5%), and e-cigarettes and/or IQOS (2.0%). The most common product ever used was CCs (94.2%), followed by nargila (52.4%), and e-cigarettes/IQOS (14.1%). Of those who had ever used CCs, 54.2% were current smokers. Of those who had ever used nargila, 22.0% were current users, and of those who had ever used e-cigarettes or IQOS, 14.0% were currently using e-cigarettes or IQOS. Of ever-nargila users, 3.9% had never used CCs or e-cigarettes. Of ever-vapers, 2.7% had never used CCs or nargila. See Supplementary File 2.

Among all respondents, 42.0% were not currently using any product, 52.1% were using a single product, 5.7% were using two products, and 0.25% were using three products. Among ever-smokers, 43.1% had only used CCs, 42.4% were dual users of CCs and nargila, 4.3% were dual users of CCs and e-cigarettes or IQOS, and 10.2% had used three products.

Among all respondents, prior to COVID-19, 44.9% used a product at least daily in their home, while 10.7% used a product in the home weekly, monthly, or less than monthly, and 44.4% had never used a product in their home.

### Changes in behavior regarding use of CCs, nargila, e-cigarettes/IQOS, and home smoking

**Fig. 1:**
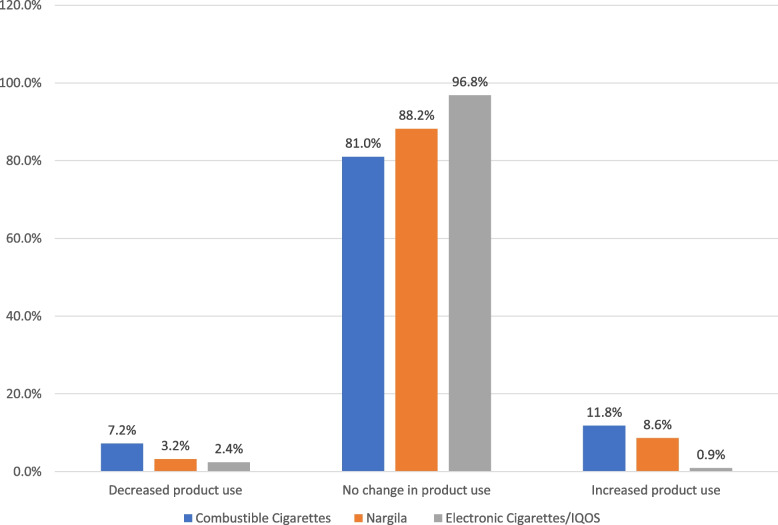
Reported changes in use of tobacco and nicotine products among Israelis following the first COVID-19 lockdown

Use of all products was unchanged in a majority of respondents (CCs: 81.0%, nargila: 88.2%, e-cigarettes/IQOS: 96.8%). While 7.2% of ever-users of CCs decreased or quit smoking, 11.8% increased the amount of their CC smoking. For nargila, the results were similar (decreased/quit: 3.2%, increased: 8.6%); 2.4% of repondents reported decreasing or quitting e-cigarette use and 0.9% reported using e-cigarettes more frequently. Figure 1 shows these changes. Table 1 presents demographic variables by change in CC smoking.

In Table 2, we present the results for changes in use of products due to COVID-19. There were no statistically significant associations between change in CC use for any of the potential explanatory variables. Non-current nargila users were significantly less likely to increase nargila use (aOR:0.06,CI:[0.02,0.21,*p* < .001) The model on changes in e-cigarette/IQOS use did not meet the convergence criterion, and therefore results are not presented.

**Table 2:** Statistical model results: Changes in product use and home smoking following Wave 1 COVID-19 Lockdown

		Change in use of combustible cigarettes, among ever-smokers of combustible cigarettes (***n*** = 259)		Change in use of nargila, among ever-users of nargila (***n*** = 164)		Change in home smoking (***n*** = 272)	
		Adjusted OR [95% CI]	***p***-value	Adjusted OR [95% CI]	***p***-value	Adjusted OR [95%CI]	***p***-value
Sex	Females vs. Males	0.90 [0.47,1.75]	0.76	0.94 [0.25,3.53]	0.93	1.24 [0.61,2.55]	0.56
Population Group	Jewish versus Arab population	0.77 [0.32,1.86]	0.56	0.43 [0.14,1.36]	0.15	0.61 [0.24,1.53]	0.29
Age	18–39 vs. 60+	1.65 [0.76,3.58]	0.32	3.28 [0.56,19.1]	0.25	1.50 [0.64,3.52]	0.65
	40–59 vs. 60+	1.05 [0.47,2.34]		1.47 [0.22,10.04]		1.34 [0.55,3.28]	
Anxiety		1.06 [0.47,2.34]	0.78	0.65 [0.34,1.26]	0.20	1.59 [1.04,2.42]	0.03
Cigarette smoking risk perceptions		0.80 [0.56,1.13]	0.20	(Not included)		1.09 [0.75,1.57]	0.66
Current product use status (For Combustibles: Current use of CCs. For Nargila: Current use of Nargila. For home smoking: Any current product use)	No vs. Yes	0.84 [0.46,1.53]	0.57	0.06 [0.02,0.21]	< 0.001	0.58 [0.30,1.14]	0.11

Data are weighted and adjusted for age, sex, and population group distributions of ever-smokers, according to data from the Israel Central Bureau of Statistics. *CI* Confidence interval. Note: Because there was a complete separation of data points when running the statistical model for e-cigarettes, and the maximum likelihood estimate did not exist, we do not present results from that model

Most respondents (83.4%) did not change their home smoking behavior due to COVID-19; However, 4.0% reported smoking less in the home, while 12.6% reported smoking more in the home. Sex, subpopulation, and age were not significantly associated with change in home smoking. Greater increases in anxiety due to COVID-19 were significantly associated with increases in home smoking (aOR: 1.59 CI:[1.04,2.42], *p* = .031) (see Table 2).

### Risk perceptions and anxiety levels

Most respondents (62.0%) believed that COVID-19 would be more severe for cigarette smokers than for non-smokers, while only 3.3% believed that COVID-19 would be lese severe. Nearly half (45.3%) believed that COVID-19 would be more severe for vapers relative to non-vapers, and just 1.6% of respondents thought that a vaper’s illness would be less severe. About a fifth of respondents (20.5%) didn’t know whether a smoker’s illness would be more severe, and 41.3% of respondents didn’t know whether a vaper’s illness would be more severe (see Supplementary File 3). Relative to males, females were more likely to believe that COVID-19 would be more severe for smokers (*p* = .003). There was an inverse relationship between risk perceptions regarding CCs and age group, with younger respondents being more likely to report that COVID-19 would be more severe for smokers than for non-smokers (*p* = 0.025). None of the explanatory variables reached statistical significance for the endpoint risk perceptions regarding vaping. Women had greater increased anxiety levels due to COVID-19 relative to men (*p* < 0.001) (see Table 3).

**Table 3:** Statistical model results: Risk perceptions and anxiety following Wave 1 COVID-19 Lockdown

		Risk perceptions: Combustible Cigarettes (*N* = 279)		Risk perceptions: Electronic Cigarettes (*N* = 198)		Anxiety (*N* = 359)	
		Least Squared Mean (LSM) +/−Standard Error (SE) / Beta +/− SE	*p*-value	LSM+/−SE / Beta +/− SE	*p*-value	LSM+/−SE / Beta +/− SE	*p*-value
**Sex**							
Female		LSM+/−SE: 1.69+/− 0.11	0.003	LSM+/−SE:1.76+/− 0.13	0.11	LSM+/−SE:3.74+/− 0.08	< 0.001
Male		LSM+/−SE: 2.02+/− 0.08		LSM+/−SE:1.98+/− 0.1		LSM+/−SE:3.33+/− 0.06	
**Population Group**	Jewish population	LSM+/−SE:1.86+/− 0.06	0.97	LSM+/−SE:1.87+/− 0.07	0.99	LSM+/−SE:3.51+/− 0.04	0.71
	Arab population	LSM+/−SE:1.85+/− 0.15		LSM+/−SE:1.87+/− 0.18		LSM+/−SE:3.55+/− 0.11	
**Age**	18–39	LSM+/−SE:2.04+/− 0.1	0.03	LSM+/−SE:1.99+/− 0.11	0.08	LSM+/−SE:3.56+/− 0.07	0.80
	40–59	LSM+/−SE:1.84+/− 0.11		LSM+/−SE:1.97+/− 0.13		LSM+/−SE:3.53+/− 0.08	
	60+	LSM+/−SE:1.69+/− 0.13		LSM+/−SE:1.65+/−0.15		LSM+/−SE:32.50+/− 0.09	
**Anxiety**	(1 = much less anxious, 5 = much more anxious)	Beta +/− SE: −.02+/−.07	0.78	Beta +/− SE:-.11+/−.08	0.15	**NR**	

*NR* Not Relevant

## Discussion

The early phase of the COVID-19 pandemic appeared to have had no net effect on changing tobacco/nicotine use behaviors among study respondents who had ever smoked, vaped, or experimented with tobacco or nicotine products in Israel.,A majority of respondents believed that smoking increased severity of COVID-19, while close to half believed that COVID-19 would be more severe for vapers relative to non-vapers Uncertainty regarding the relationship between smoking CCs, vaping, and COVID-19 was common, likely reflecting the scientific ambiguity and the conflicting messages from various scientific reports and from health organizations, in Israel and abroad. Uncertainty about vaping was considerably higher than uncertainty about smoking. Most respondents did not change their home smoking behavior due to COVID-19; however, home-smoking was more likely to increase among those who experienced greater anxiety because of COVID-19.

We found that there was no substantial net change in smoking among our sample of current and ex-smokers (7.2% decreased or quit smoking and 11.8% increased the amount of their smoking). This dual trend of both increasing and decreasing smoking has also been observed in numerous other countries. For example, a nationally representative online study of cigarette smokers conducted in the Netherlands reported that 18.9% of cigarette smokers increase their smoking, and 14.1% decreased their smoking. A web-based US study found that 30.3% increased and a 28.3% decreased smoking. Another online study in the US found that 24% of smokers increased their consumption and 28.0% decreased smoking [44]. A study of 6870 adult smokers conducted in Australia, Canada, England, and the US found that 1.1% attempted to quit, 14.2% reduced smoking, and 14.6% increased smoking (70.2% reported no change) [28]. A national study conducted in Canada found that 3% increased their smoking and 2% decreased their smoking [45]. The study conducted in Israel by Bar-Zeev et al. [31] found that 44.3% of current smokers increased their consumption. This contrasts with our study, which found a much lower proportion of respondents who increased their CC use (11.8%). However, this may be due to differences in sample composition: we included ever-smokers, while Bar-Zeev et al. assessed changes in smoking among current smokers.

Although there are many studies that have examined changes in smoking among adults during COVID-19, few have examined changes in vaping behaviors. A UK study conducted online between April 2020 and June 2021 found that 25% reported a quit attempt (16% due to COVID-19-related reasons) and the quit rate was 18%. At 12 months, 48% of continuing vapers reported no change in their vaping frequency, while similar proportions reported vaping less (27.5%) and more (24.8%) [46]. Another UK study reported that a minority (12.2%) of quit attempts in the past 3 months were reportedly triggered by COVID-19, and approximately one in ten current e-cigarette users reported attempting to quit vaping because of COVID-19 [47]. In the US, an April–June 2020 study using data collected via Mechanical Turk found that 27.3% of e-cigarette users had increased vaping since the start of the pandemic and 23.8% had decreased (the remaining vapers did not change their consumption) [44]. We found a similar trend whereby the majority did not change their vaping frequency (2.4% of respondents reported decreasing or quitting e-cigarette use and 0.9% reported increasing their use). Our estimates regarding e-cigarettes/IQOS are low, likely due to the fact that few people use e-cigarettes relative to the UK and US.

One possible explanation for this dual trend lies in the increased levels of anxiety felt by smokers due to the coronavirus outbreak. Anxiety could cause some individuals to smoke more, and others to smoke less. Before the pandemic, anxiety was found to be a significant factor in increasing the readiness to quit smoking in Arab males in Israel. The connection between this dual trend and anxiety is shown in a Dutch study that found a dose-dependent relationship between stress and both increased and decreased smoking rates [48].

One source of anxiety may be due to the higher rates of unemployment seen during COVID-19, which caused significant financial stress even in countries such as Israel, which had a strong economic safety net both before and during COVID-19. We found that 41.7% of Israelis reported experiencing increased anxiety due to the pandemic. This is hardly an isolated phenomenon: a study in Germany conducted in late March and early April 2020 found that over 50% of participants experienced suffering from anxiety and psychological distress due to the pandemic, and a study in India found that 40% of respondents reported worry, anxiety, and paranoia at the thought of contracting the virus [49]. Furthermore, a study in Ireland found that generalized anxiety disorder and depression were associated with loss of income during the pandemic and higher levels of perceived risk from COVID-19 [50]. A systematic review found that the general public experienced lower psychological wellbeing and higher levels of anxiety and depression during the pandemic [51]. In our study, although we did not find a statistically significant relationship between anxiety and changes in CC or nargila smoking, the elevated anxiety rates we observed may still be cause for concern since increased anxiety levels are associated with higher smoking consumption, higher rates of COPD and lung neoplasms, and stress has been shown to play a role in perpetuating drug use and relapse [52–55].

It should be noted that the lack of a net effect of COVID-19 on smoking constitutes a less negative population-level impact than major population crises such as natural disasters or terrorist attacks like 9/11 [56, 57]. This may be a consequence of the public health messaging in Israel and internationally that smoking is linked to more severe COVID-19 illnesses, perhaps amplifying the intuitively appealing notion that a respiratory illness would be more severe among smokers. The explicit link between the source of the crisis and smoking is unique to COVID-19, relative to other population-level disasters, which may explain why COVID-19 did not lead to increased smoking.

The pandemic caused many people to spend an increased amount of time indoors, whether because of policy aimed at reducing transmission, fear of infection, or suspension of regularly scheduled activities in schools, social organizations, and places of business. We found that, prior to COVID-19, 55.6% of respondents used tobacco or nicotine products in their homes, with 12.6% of smoking respondents reporting an increase in their frequency of home product use due to coronavirus. Changes in home smoking were significantly associated with anxiety; those respondents more anxious about COVID-19 were more likely to increase their smoking in the home. This is a cause for concern as indoor smoking is causally linked to lung cancer, stroke, and cardiovascular disease in adults and low birth weight, sudden infant death syndrome, ear infections, and asthma in children [58]. Furthermore, nonsmoking occupants, particularly children, are spending more time at home, and thus increasing their exposure to pollutants. Even before the pandemic, based on research in 31 countries, 78% of women and children living with a smoker, and 59% of women and young children not living with a smoker, had biomarkers demonstrating tobacco smoke exposure [59].

Nargila smoking, which produces large amounts of smoke, has been found to cause significantly higher levels of carbon monoxide, both in the room where the smoking occurs in and in adjacent rooms [60]. Previous research showed that Arab infants had a high frequency of exposure to tobacco smoke (52%), as well as high levels of exposure of Jewish infants (Jews: 24.8%) [61]. Interventions are sorely needed to convince people to refrain from using tobacco products in the home, and especially to protect “captive” non-smoking women and children who are exposed to second-hand tobacco smoke in their own homes.

Strengths and Limitations: One of the strengths of this study was that it was based on a representative sample of Israeli households, including majority (Jewish) and minority (Arab) populations, and was conducted in four languages: Hebrew, Arabic, Russian, and English. Moreover, the weighted analyses allowed for generalizations to the population of Israel. In addition, the study measured a variety of inhaled tobacco and nicotine products (cigarettes, nargila, e-cigarettes, IQOS). Because most participants did not change their behavior following the first COVID-19 lockdown, we had low power to identify correlates of change, leading to our inability to detect small differences in behavior between the majority versus the minority populations. Small numbers of e-cigarette/IQOS users precluded assessment of the association between potential explanatory variables and change in e-cigarette/IQOS use. When asking about smoking in the home, we did not differentiate between types of products used, in addition, anxiety was measured using a single question and was self-assessed by respondents. The study was conducted during the initial phase of the pandemic and it is not clear whether the findings are applicable to the subsequent waves of COVID-19 that followed.

## Summary

Our findings demonstrate a need for: (1) provision of clear evidence-based information on what is known about the relationship between smoking, vaping, and COVID-19, and (2) strong messaging to deter home smoking which may increase as stress increases. This is especially important for household residents who are members of vulnerable populations, particularly young children, pregnant women, and the elderly. Government-sponsored messages could be realistically provided, at nearly no cost to the government, via mandated but as yet unimplemented tobacco package inserts, whose costs would be incurred not by government, but by tobacco and nicotine companies [62].

## Supplementary Information

**Additional file 1.** Weighting.

**Additional file 2.** Reported use of combustible cigarettes (CCs), Nargila, and E-cigarettes/IQOS before COVID-19, by Population Group and gender: Descriptive statistics (Weighted data).

**Additional file 3.** Perceptions of increased risk of COVID-19 severity for combustible cigarette smokers and vapers.

## Data Availability

The datasets generated and analyzed during the current study are not publicly available due to the fact that we did not ask our participants for permission to make the data publicly available. Anonymized data (with no identifying information about the respondents) are available from the corresponding author on reasonable request.

## Abbreviations

CC’s: Combustible cigarettes
IQOS: A heated tobacco product produced by Philip Morris
CDC: Center for Disease Control
WHO: World Health Organization
HMO: Health Maintenance Organization
CBS: Israeli Central Bureau of Statistics
ITC: International Tobacco Control Policy Evaluation Project
