# Supplementary material for: The association between risk perceptions, anxiety, and self-reported changes in tobacco and nicotine product use due to COVID-19 in May-June 2020 in Israel
Source: BMC Public Health. 2023 Apr 25;23:759. doi: 10.1186/s12889-023-15351-1 (PMC10126559; doi:10.1186/s12889-023-15351-1)
Supplement: Supplementary file 1 — Additional file 1. Weighting. [file 12889_2023_15351_MOESM1_ESM.docx]

**Supplementary File 1: Weighting**

Data were weighted for factors gender (male/female), Population Group (Jews&Others, Arabs), and age category (18-39, 40-59, 60+). Joint distributions of gender, Population Group, and age category from the Israeli population of current of former smokers (including cigarettes and nargila users) were obtained from the Central Bureau of Statistics (CBS) Table Generator (1) Because we were able to obtain data on current and former smokers who were 20 years and older, but our study also included 18-19 year olds, we increased the numbers obtained from the CBS for the age group 20-39 by 10% (estimated from the actual population of 18-19 year olds relative to other adults) before creating the weights.

The original distributions from CBS were as follows:


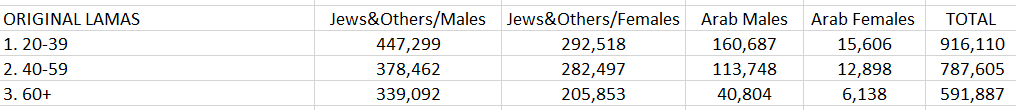


The distributions with the additional 10% were as follows:

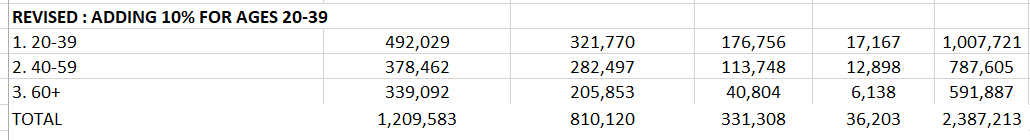


Cell percentages from the CBS data were as follows:


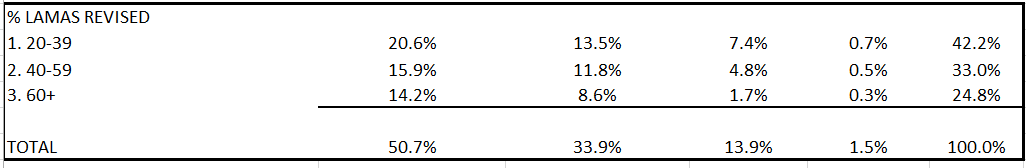


Cell percentages from out survey data:


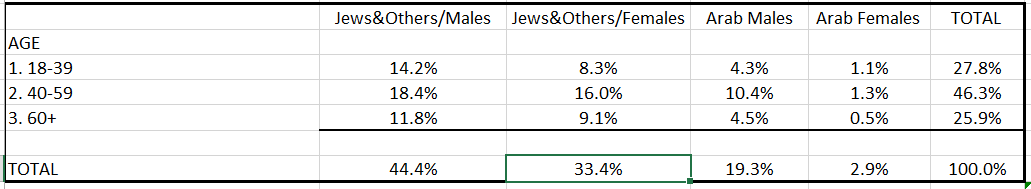


The final weights were calculated as cell percentages from revised CBS data divided by cell percentages from our survey data:


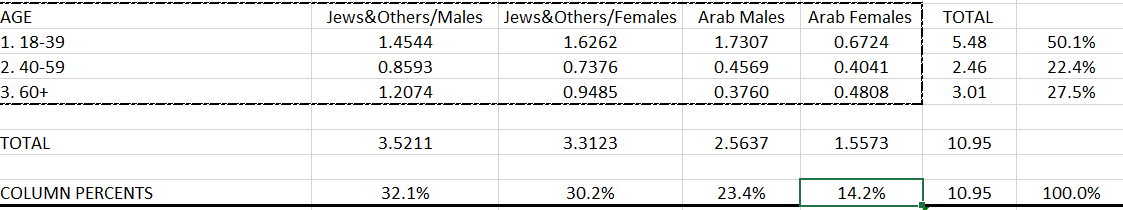


1. Israel Central Bureau of Statistics. Social Survey Table Generator 2017 [Available from: <https://www.cbs.gov.il/en/Pages/default.aspx>. Accessed Jan. 13, 2023.
