# Supplementary material for: The association between risk perceptions, anxiety, and self-reported changes in tobacco and nicotine product use due to COVID-19 in May-June 2020 in Israel
Source: BMC Public Health. 2023 Apr 25;23:759. doi: 10.1186/s12889-023-15351-1 (PMC10126559; doi:10.1186/s12889-023-15351-1)
Supplement: Supplementary file 2 — Additional file 2. Reported use of combustible cigarettes (CCs), Nargila, and E-cigarettes/IQOS before COVID-19, by Population Group and gender: Descriptive statistics (Weighted data). [file 12889_2023_15351_MOESM2_ESM.docx]

**Supplementary File 2: Reported use of combustible cigarettes (CCs), Nargila, and E-cigarettes/IQOS before COVID-19, by Population Group and gender: Descriptive statistics (Weighted data)**

|  |  | **Combustible Cigarettes** | **Nargila** | **E-cigarettes/IQOS** |
| --- | --- | --- | --- | --- |
| **Jewish population - males** | Current | 52.0% | 12.3% | 3.9% |
|  | Former | 43.4% | 24.0% | 12.8% |
|  | Experiment | NA | 18.2% | 3.0% |
|  | Never | 4.7% | 45.6% | 80.3% |
| **Jewish population - females** | Current | 47.4% | 3.2% | 0% |
|  | Former | 43.8% | 14.9% | 3.3% |
|  | Experiment | NA | 22.7% | 4.3% |
|  | Never | 8.7% | 59.3% | 92.4% |
| **Arab population - males** | Current | 55.5% | 29.6% | 0% |
|  | Former | 41.8% | 29.8% | 5.1% |
|  | Experiment | NA | 13.8% | 5.1% |
|  | Never | 2.7% | 26.8% | 89.7% |
| **Arab population - females** | Current | 62.8% | 7.1% | 0% |
|  | Former | 29.6% | 11.9% | 8.5% |
|  | Experiment | NA | 38.0% | 0% |
|  | Never | 7.7% | 43.1% | 91.5% |
| **All** | Current | 51.0% | 11.5% | 2.0% |
|  | Former | 43.1% | 21.5% | 8.4% |
|  | Experiment | NA | 19.4% | 3.7% |
|  | Never | 5.9% | 47.6% | 85.9% |

**NA: Not Available**
