# Supplementary material for: The association between risk perceptions, anxiety, and self-reported changes in tobacco and nicotine product use due to COVID-19 in May-June 2020 in Israel
Source: BMC Public Health. 2023 Apr 25;23:759. doi: 10.1186/s12889-023-15351-1 (PMC10126559; doi:10.1186/s12889-023-15351-1)
Supplement: Supplementary file 3 — Additional file 3. Perceptions of increased risk of COVID-19 severity for combustible cigarette smokers and vapers. [file 12889_2023_15351_MOESM3_ESM.docx]

**Supplementary File 3: Perceptions of increased risk of COVID-19 severity for combustible cigarette smokers and vapers**

| **Population Group** | **Risk perception*** | **Risk to Combustible Cigarette Smokers** | **Risk to Vapers** |
| --- | --- | --- | --- |
|  |  | Weighted estimate (95% CI) | |
| **Jewish population - males** | More severe | 57.0% | 37.8% |
|  | Same | 15.4% | 13.7% |
|  | Less severe | 3.4% | 1.8% |
|  | Don’t know | 24.2% | 46.7% |
| **Jewish population - females** | More severe | 73.9% | 61.4% |
|  | Same | 11.0% | 8.3% |
|  | Less severe | 3.3% | 0.6% |
|  | Don’t know | 11.8% | 29.8% |
| **Arab population - males** | More severe | 47.5% | 33.0% |
|  | Same | 19.1% | 14.7% |
|  | Less severe | 3.3% | 3.3% |
|  | Don’t know | 30.1% | 49.0% |
| **Arab population - females** | More severe | 91.5% | 46.4% |
|  | Same | 0.0% | 0.0% |
|  | Less severe | 0.0% | 0.0% |
|  | Don’t know | 8.5% | 53.6% |
| **All** | More severe | 62.0% | 45.3% |
|  | Same | 14.2% | 11.8% |
|  | Less severe | 3.3% | 1.6% |
|  | Don’t know | 20.5% | 41.3% |

***Questions:**

1 - Thinking about smokers in general, if a smoker got the coronavirus, how severe do you think the illness would be for them, compared to non-smokers of the same age who got it? A lot more severe than for non-smokers of the same age/ Somewhat more severe/ Neither more nor less severe/ Somewhat less severe/A lot less severe/ Refused/Don’t know

2 - Thinking about electronic cigarette users and vapers, those who do not also smoke cigarettes, if a vaper got the coronavirus, how severe do you think the illness would be for them, compared to non-users of electronic cigarettes or vaping products of the same age who got it? A lot more severe than for non-smokers of the same age/ Somewhat more severe/ Neither more nor less severe/ Somewhat less severe/A lot less severe/ Refused/Don’t know
